# Increasing the proportion of healthier foods available with and without reducing portion sizes and energy purchased in worksite cafeterias: protocol for a stepped-wedge randomised controlled trial

**DOI:** 10.1186/s12889-019-7927-2

**Published:** 2019-12-02

**Authors:** James P. Reynolds, Daina Kosīte, Brier Rigby Dames, Laura A. Brocklebank, Mark Pilling, Rachel Pechey, Gareth J. Hollands, Theresa M. Marteau

**Affiliations:** 1 Behaviour and Health Research Unit, University of Cambridge, Cambridge, UK; 2 School of Psychological Science, University of Bristol, Bristol, UK

**Keywords:** Physical micro-environment interventions, Choice architecture, Nudging, Stepped wedge trial, Randomised controlled trial, Healthier eating, Obesity, Workplace interventions, Portion size, Availability

## Abstract

**Background:**

Overconsumption of energy from food contributes to high rates of overweight and obesity in many populations. A promising set of interventions tested in pilot studies in worksite cafeterias, suggests energy intake may be reduced by increasing the proportion of healthier *–* i.e. lower energy – food options available, and decreasing portion sizes. The current study aims to assess the impact on energy purchased of *i.* increasing the proportion of lower energy options available; *ii.* combining this with reducing portion sizes, in a full trial.

**Methods:**

A stepped-wedge randomised controlled trial in 19 worksite cafeterias, where the proportion of lower energy options available in targeted food categories (including main meals, snacks, and cold drinks) will be increased; and combined with reduced portion sizes. The primary outcome is total energy (kcal) purchased from targeted food categories using a pooled estimate across all sites. Follow-up analyses will test whether the impact on energy purchased varies according to the extent of intervention implementation.

**Discussion:**

This study will provide the most reliable estimate to date of the effect sizes of two promising interventions for reducing energy purchased in worksite cafeterias.

**Trial registration:**

The study was prospectively registered on ISRCTN (date: 24.05.19; TRN: ISRCTN87225572; doi: 10.1186/ISRCTN87225572).

## Background

Unhealthy patterns of food consumption, including excess energy intake, contribute to high and rising rates of obesity worldwide (GBD [1–3]), which leads to increasing incidences of type 2 diabetes and 13 different types of cancer [4]. Providing information about obesity to the public is a popular strategy [5], but has little or no effect at changing patterns of behaviour at the scale needed [6, 7]. In part, this reflects the powerful impact of environmental cues that shape unhealthier behaviours, regardless of intentions to act differently [8, 9]. Changing these environmental cues is, therefore, key to achieving the scale of behaviour change needed to tackle obesity [10, 11].

Three Cochrane reviews highlight the potential of three sets of interventions that involve cues in the physical micro-environment: increasing the availability of healthier foods [12], reducing portion sizes [13], and adding calorie labelling [14]. These reviews also draw attention to the paucity of high-quality studies that have been conducted in real-world settings. To address this, a series of pilot studies were conducted to assess the impact on energy purchased in worksite cafeterias of these three interventions [15], a particularly important environment to target for interventions as an estimated 11–18% of an adult’s meals occur while at work [16]. The results provide preliminary evidence for the effectiveness of the availability and portion size interventions [17, 18], but not for labelling interventions [19, 20].

There is currently a small evidence base for availability interventions – only six studies met the inclusion criteria for the Cochrane review assessing impact on selection or consumption of altering the availability of food, alcohol or tobacco products [12]. Providing fewer options of a targeted food or food category led to a large reduction in their selection but with wide confidence intervals. One of the studies in the Cochrane review was the pilot study in worksite cafeterias, which resulted in a reduction of 7% in energy (kcal) purchased per day from the targeted food categories across six cafeterias, with no significant impact on revenue [18]. A similar effect was seen in a recent field study increasing the availability of vegetarian meal options (and decreasing the availability of meat ones) on selection in student cafeterias [21].

In terms of interventions targeting portion sizes, there is substantial experimental evidence that portion size reductions reduce selection and consumption of food [22, 23]. The Cochrane review that investigated size interventions included 69 studies in food, estimating that consistently reducing portion sizes could reduce energy intake by up to 16% in UK adults [13]. It also highlighted some key outstanding uncertainties, in particular the relative absence of evidence from real-world settings and over sustained time periods. One concern with the real-world application of this intervention is that customers are more likely to choose larger portions when larger options are available, thereby potentially undermining this intervention if changes are not applied to every available product. A further concern is that consumers may compensate by purchasing more products if they perceive that their portion is too small. Some evidence suggests that, contrary to a compensatory mechanism, portion size reductions may change related norms, leading consumers to purchase smaller food items in the future [24, 25], but it is unclear if this effect is generalisable; particularly to those with physically tiring jobs. A recent pilot trial in six worksite cafeterias reduced the portion size of main meals, sides, desserts, and cakes by approximately 10–15%, which led to a statistically non-significant but numerical reduction in energy purchased from intervention categories of approximately 8% [17]. However, larger field trials that recruit more sites and test interventions for longer periods of time are needed to provide more precise estimates for the effectiveness of both availability and portion size interventions.

The proposed study will provide the most reliable estimate to date of the effect sizes of two promising interventions for reducing energy purchased in 19 worksite cafeterias: increased proportion of lower energy foods available for selection, assessed on its own and in combination with reduced portion sizes. The cafeterias are located across Great Britain, serving mainly manual workers, a group with typically poorer diets, as reflected in their higher rates of obesity, type 2 diabetes and associated cancers [4]. Manual workers therefore have more to gain than non-manual workers from effective interventions. Study hypotheses: 1. fewer calories will be purchased during the availability intervention period when compared to the baseline; 2. fewer calories will be purchased during the availability + portion size intervention period when compared to the baseline and when compared to the availability intervention period.

## Methods

### Study design

A stepped-wedge design (Campbell & Walters, 2014) will be used, with each of the 19 sites randomly allocated to the time at which they implement each of the two interventions across a period of 25 weeks (see Additional file 2). This design was selected over a parallel groups cluster RCT due to insufficient resources to implement the intervention(s) at all sites simultaneously. Weeks 1 to 4 will comprise the minimum baseline period – during which data will be recorded without any intervention – followed by 21 weeks during which interventions will be introduced and maintained. From Week 5 until Week 13, two sites a week will implement the first intervention – Availability. In Week 14, the 19th site will implement this intervention. From Week 13 until Week 21, two sites a week will implement the second intervention – Size – while continuing the Availability intervention. In Week 22, the 19th site will implement this intervention. The interventions will continue until the end of Week 25 for all sites (the duration of the Size intervention in sites will therefore vary between 4 and 13 weeks).

The study was prospectively registered on ISRCTN (10.1186/ISRCTN87225572). Any significant modifications to the study will be updated on this registration.

### Interventions

#### Increased availability of healthier foods (Availability)

Availability interventions applied to products can be conceptualised into three broad categories: changing the relative availability of products, changing the absolute availability of products, or changing both [26]. The intervention in the current study comprises increasing the number of healthier food options and decreasing the number of less healthy food options to maintain the same total number of options (i.e. changing the relative availability). In the TIPPME intervention typology [10] it is classified as an Availability x Product intervention.

In the current study, healthiness is defined by energy content, with the term ‘healthier’ indicating lower energy (kcal) foods according to cut-off points varying by product type (e.g., main meal, cold drink, etc.) (see Additional file 1). Exceptions include fruit, vegetables, nuts and seeds without added sugar or salt, and 100% fruit juice, which are classed as healthier regardless of energy content. Energy content is not the only factor in determining the healthiness of a specific food item, but we have selected it because excess energy is a major contributor to population-level excess weight and obesity. It also enables an unambiguous and quantifiable scale of healthiness. The proposed implementation of this intervention is similar to that of our pilot trial [18]. A visual representation of the Availability intervention is provided in Additional file 4.

The target proportion of less healthy/healthier items varies by intervention category and is dependent on the availability of lower energy options that can be procured and introduced by the companies that provide the food and drink to the cafeterias. Where possible, the highest energy options will be selected to be removed, to maximise the effectiveness of the intervention (see Additional file 1). Items that are removed will be replaced with similar products that have a calorie content below the cut-off for the respective food category (see Additional file 1).

#### Reduced portion size (Size)

This intervention comprises reducing the portion size, by volume, of products in the targeted food categories (see Additional file 1). Within the TIPPME intervention typology [10] this is classified as a Size x Product intervention.

Within targeted food categories, changes will be requested only for products classified as less healthy using the cut-offs listed in Additional file 1. These will include – but not be limited to – all products that are served in trays (e.g. pies), countable in pieces (e.g. scampi), wet/served with a ladle (e.g. curry, rice) or sliced or portioned by the sites (e.g. cakes), as these enable reductions to be made most readily and precisely. The reductions in portion size will vary by site and specific product but will be requested to be at least a 10% reduction by volume in each targeted product. We will also request that any reduction in portion size is accompanied by an equivalent change in price.

The Availability intervention will be implemented and evaluated on its own. Following this, the Size intervention will be added to evaluate the combined effect of these two interventions. This design was selected for two reasons. First, it allows us to assess Availability in isolation, as the pilot studies indicated this was the more effective intervention, before then assessing the combined effect. Second, it ensures that the foods offered are similar across the two interventions, because implementing the Availability intervention involves altering some of the foods offered, unlike the Size intervention.

#### Fidelity checks

Researchers will carry out visits to each worksite cafeteria to monitor the baseline food offer (one visit), the implementation of the Availability intervention (two visits), and the implementation of the Size + Availability intervention (two visits). If concerns are raised about the implementation of an intervention during this visit the following occurs:

i. The researcher sends a detailed description of her/his concerns to the study manager (JR);
ii. The catering manager for the site is contacted with a view to addressing the concern that working day;
iii. A follow-up visit is arranged - if possible – within two working days.

These visits along with regular communications with a manager at each site are also conducted to promote site retention and engagement.

#### Blinding

Cafeteria staff will be aware of the nature of each intervention to enable the implementation of the interventions. Customers will be told via posters that a health initiative is being implemented to improve the healthiness of the food and drink on offer in the cafeteria. They will not be informed about the actual changes.

### Targeted food categories for availability and/or portion size interventions

The precise food categories and products within those that receive each of the interventions will depend on discussions with cafeteria managers and catering companies but will likely include the following:

- *Main meals*: meat or vegetarian principal element of a meal
- *Sides*: carbohydrate rich portions (e.g.*,* chips)
- *Desserts:* hot desserts (e.g.*,* crumbles), dessert pots (e.g.*,* yoghurt, cheesecake, mousse, jelly, granola) and sliced cake
- *Bakery*: freshly made cakes, muffins, cookies, pre-packed croissants and flapjacks
- *Savoury snacks:* e.g.*,* crisps
- *Confectionery*: e.g.*,* chocolate bars, sweets
- *Cold drinks*: e.g.*,* cans of sugar sweetened beverages, bottles of water
- *Sandwiches:* pre-packaged sandwiches, baguettes, panini

Intervention categories are defined as a category of food or drink in which we had formal agreement to change the product range or product size as part of the Availability or Size interventions. Categories that we anticipate will not receive the interventions include soups, breakfasts, and items in vending machines.

### Setting

#### Participants and recruitment

We assessed eligibility of 29 cafeterias based in supermarket distribution centres. Two sites were excluded due to difficulty of access, two were excluded due to employing fewer than 350 workers, and a further six were excluded due to practical problems with the consistent mapping of the electronic till buttons (see Additional file 3).

These exclusions resulted in 19 eligible sites, all of which agreed to take part in the study. Each of these sites employ between 530 and 1453 workers and are based in England, Scotland, or Wales. All sites belong to the same UK-based supermarket chain and are managed by one of three separate catering companies.

#### Inclusion criteria

- The cafeteria is based in a distribution centre that belongs to the UK-based supermarket chain that we have partnered with for this project
- The distribution centre has at least 350 employees
- The cafeteria has Electronic Point of Sale tills which can record sales data electronically

#### Exclusion criteria

- Not meeting the inclusion criteria
- Site is too difficult to access (requiring travel by air or sea)

#### Sample size determination

The sample size calculation was based on two pilot previous studies [17, 18]. We conservatively used the largest estimate of standard deviation (0.111) when analysis was on the log scale. A one-sided t-test at 80% power with 5% significance level and *n* = 19 cafeterias would be able to detect an effect size of 6.5% or greater reduction in energy purchased (i.e. equivalent to a reduction of − 0.067 on the log scale). We selected a one-sided test, reflecting existing evidence. This includes previous worksite studies of the same interventions to be evaluated in the current study – Availability and Size [17, 18] – in which there were reductions in 11 of 12 cafeterias using the same primary outcome as will be used in the current study – i.e. energy purchased in intervention categories. This overall direction of effect is also evident in the findings of Cochrane reviews of each of these two interventions: both interventions reduce selection of less healthy foods [12, 13].

The study duration was balanced against pragmatic considerations which results in a 25-week design with up to two sites randomised to an intervention each week.

#### Withdrawal of participants

Further power calculations suggest that if any cafeterias drop out then *n* = 14 cafeterias would detect an effect size of 8%, *n* = 11 for 9%, and *n* = 10 for 10%.

### Randomisation

After all the sites had agreed to participate, randomisation was performed by a blinded statistician (MP) who allocated a list of anonymised site names using the rank of random numbers from Excel.

### Measures

#### Primary outcome

Total energy (kcal) purchased from intervention food categories per day, calculated from the total number of units sold of each individual product within an intervention food category and the total number of calories contained in each of these products. Sales data are recorded using electronic tills every day of operation during the trial. These data are then sent electronically to the study team.

#### Secondary outcomes

i. Total energy (kcal) purchased per day from

- non-intervention categories, and
- all food and drink products.

ii. Total revenue

This is calculated from the number of units of each individual product sold in the cafeterias and the price of each of these products. These data will be collected using the electronic tills for every day of operation during the trial.

#### Covariates

Analyses (with the exception of those examining revenue) will control for the total number of daily transactions to take account of footfall. Transactions are defined as the number of unique payments to purchase products in the cafeteria.

Further variables may include the catering company serving the distribution centre, the day of the week, weather conditions at the location (daily mean temperature, daily hours of sunshine, and daily rainfall), whether calorie information is displayed for the products on offer, and notable events (e.g., a workplace party) that may affect sales.

### Additional measures

Demographic characteristics of employees, including age, gender, and occupational status, will be requested for each distribution centre.

Variables relevant to the specific interventions will be recorded, including:

*For the Availability intervention*: proportion of food/drink intervention items that are categorised as healthier pre-intervention; proportion of food/drink items that are targeted by the intervention; mean energy (kcal) per item pre-intervention; reduction (%) in proportion of intervention items that are categorised as less healthy from pre-intervention; reduction (%) in energy (kcal) per item from pre-intervention; mean price pre-intervention; mean change (%) in price.

*For the Size intervention*: mean energy (kcal) per product pre-intervention; mean reduction (%) in energy (kcal) per product; mean price pre-intervention; mean reduction (%) in price.

### Data analysis / statistical plan

Generalised linear mixed models will be used to estimate the potential impact of the Availability intervention and the combined Availability + Size intervention compared to baseline. The primary analysis will determine the effectiveness of these interventions across all sites, with follow up tests to determine if the effect varied across sites. Primary analyses will be on an intention to treat basis (i.e. data will be analysed according to the period that they should be in [baseline, Availability, or Availability + Size], regardless of adherence to the intervention).

Any outliers will be identified using range checks, scatter plots, and histograms. If any outliers (defined as any value that differs from the median by more than 3 standard deviations) are detected then further checks will be performed by the research team to ensure they are not the result of data entry errors. The cafeterias will also be contacted to determine if there were any events that could explain these outliers as genuine or uncharacteristic. Any true outliers will be included in the primary analysis but, if deemed necessary, may be modelled by a factor if there is a regular event, and a sensitivity analysis will be completed without any true outliers to compare the robustness of model results.

Sensitivity analyses will be conducted using a per protocol analysis, i.e. data will be analysed based on adherence to the intervention; intervention categories that have failed to receive sufficient changes will be excluded from the primary outcome. Non-adherence is defined as a failure to make the pre-specified changes to an intervention category in at least 50% of the sites within the intervention week. Checks are performed by a member of the research team visiting the sites to determine whether the sites adhere to the interventions. The pre-specified threshold for intervention adherence (i.e. proportion of less healthy items offered for Availability) within each intervention category is defined in Additional file 1, with at least a 10% reduction by volume in each targeted product required for Size. If a site fails an intervention check for one category during a site visit, but then resolves the issue within the same week (Monday-Sunday), then the site will be recorded as passing that intervention checking.

Due to the nature of the stepped-wedge design it will not be possible to blind the statistician to intervention status while conducting the analysis.

## Discussion

This study will provide the most reliable estimate to date in real-world settings (namely worksite cafeterias) of the effect sizes of two promising interventions for reducing energy purchased: increased proportion of lower energy foods available for selection, assessed on its own and in combination with reduced portion sizes. It builds on the results of two previous pilot studies suggesting that when implemented individually, increasing the proportion of healthier foods available could reduce energy purchased by 7% and reducing portion sizes by about 10% could reduce energy purchased by 9%, although the latter effect was not statistically significant [17, 18].

These interventions have the potential not only to tackle overweight and obesity in the workplace, but also to reduce inequalities in health due to higher rates of overweight and obesity in more deprived populations ([27]; Public Health [28]). The majority of employees that use the cafeterias included in the current study work in manual and/or unskilled roles. If this study demonstrates that these interventions can reduce energy purchased amongst such a group, which is at higher risk for ill health associated with obesity, then these interventions may be viable options for government policies to reduce ill health equitably [29].

One limitation of the current approach is that the primary outcome is estimated from sales data. While this outcome provides reliable data on sales, it is an imperfect measure of energy actually consumed. Energy consumed from other sources while at work – such as from vending machines or from food brought from home – will not be captured. It is also does not account for the incomplete consumption of food and drink purchases.

## Conclusion

This study will be the largest field study to date to estimate the impact on energy purchased of two interventions: increasing the proportion of lower energy foods available in worksite cafeterias with and without also reducing portion sizes. These are scalable interventions that could be applied in many other settings and countries, with the potential to contribute to global efforts to tackle high and rising rates of overweight and obesity.

## Supplementary information

**Additional file 1: Table S1.** Table displaying the planned implementation of the availability intervention.

**Additional file 2: Figure S1.** Figure displaying the planned stepped-wedge study design.

**Additional file 3: Figure S2.** Figure displaying the enrolment, allocation, and analysis of sites.

**Additional file 4: Figure S3.** Figure representing how a range of main meals on a single day may change between baseline and Availability periods.

**Additional file 5.** Consent form.

## Data Availability

The datasets generated and analysed during the current study cannot be made available beyond the research team as they are commercially sensitive, and provided by the participating worksites on condition that they are not shared beyond the research team.

## Abbreviations

ISRCTN: International Standard Randomised Controlled Trials Number
KCAL: 1000 cal
RCT: Randomised Controlled Trial
TIPPME: Typology of Interventions in Proximal Physical Micro-Environments
