# Supplementary material for: Increasing the proportion of healthier foods available with and without reducing portion sizes and energy purchased in worksite cafeterias: protocol for a stepped-wedge randomised controlled trial
Source: BMC Public Health. 2019 Dec 2;19:1611. doi: 10.1186/s12889-019-7927-2 (PMC6889705; doi:10.1186/s12889-019-7927-2)
Supplement: Supplementary file 1 — Additional file 1: Table S1. Table displaying the planned implementation of the availability intervention. [file 12889_2019_7927_MOESM1_ESM.docx]

Additional file 1: Table S1. Planned implementation of the availability intervention

| Categories | Items | Cut-off | Less healthy | Healthier | Proportion of less healthy options during baseline^1^ | Proportion of less healthy options during availability intervention^2^ |
| --- | --- | --- | --- | --- | --- | --- |
| Cooked main meals (excluding breakfast) | Complete main meals | 530+ kcals | e.g., roast leg of pork, stuffing, apple sauce, roast potatoes & carrots; vegetarian bolognaise with chef’s salad & garlic bread | e.g., smoked haddock kedgeree with chef’s salad; broccoli & stilton quiche with chef’s salad | 75-100% | 50% |
|  | Main meal without a side | 330+ kcals | e.g., battered fillet of pollock; BBQ slaw chicken burger | e.g., grilled salmon | 75-100% | 50% |
|  | Sides | 10%+ fat | e.g., chips; roast potatoes; buttered noodles | e.g., boiled potatoes; basmati rice | 75%* | 25%* |
| Sandwiches or equivalents | Sandwiches, wraps, baguettes | 350+ kcals | e.g., sausage sandwich on white bread | e.g., ham sandwich on brown bread | 75% | 50% |
| Snacks | Savoury snacks | 120+ kcals | e.g., full-fat thick cut crisps | e.g., popped crisps; selected popcorns | 90% | 75% |
|  | Sweet snacks | 150+ kcals | e.g., standard chocolate bars; biscuits | e.g., selected cereal bars; rice cakes | 90% | 80% |
| Drinks | Cold drinks (100% fruit juice and smoothies excluded) | 50+ kcals | e.g., full sugar soda; juice-based drinks with added sugar; energy drinks | e.g., water; diet soda; diet energy drinks | 75% | 50% |
| Dessert | Dessert pots, hot desserts | 310+ kcals | e.g., chocolate sundae; lemon sponge | e.g., jelly; French apple and pear tart | 100% | 50% |
| Bakery |  | 513 kcals (median split) | e.g., classic Victoria sponge | e.g., marmalade cake | 100% | 50% |

*Note.* ^1^ These are estimates only and will vary site to site. ^2^ This is the planned implementation only. The exact implementation will likely vary site to site. *The planned intervention is to only have 1 less healthy food option available. 25% illustrates the intervention in the scenario when four sides are available.
