# Supplementary figures and images for: Increasing the proportion of healthier foods available with and without reducing portion sizes and energy purchased in worksite cafeterias: protocol for a stepped-wedge randomised controlled trial
Source: BMC Public Health. 2019 Dec 2;19:1611. doi: 10.1186/s12889-019-7927-2 (PMC6889705; doi:10.1186/s12889-019-7927-2)

Additional file 2: Figure S1. Study design
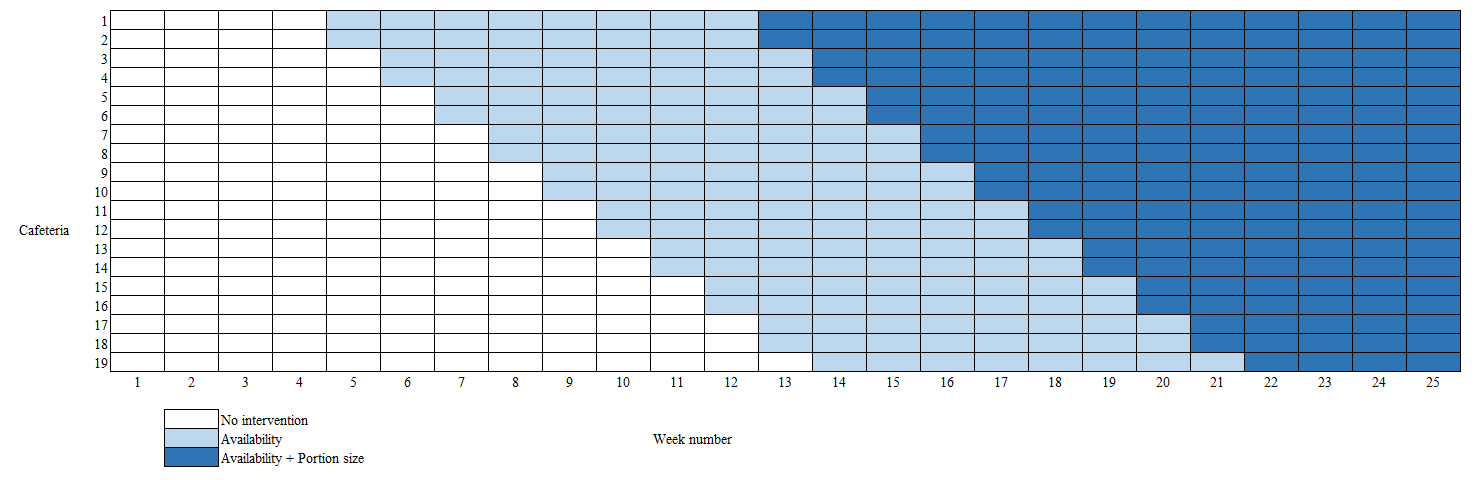

Supplement: Supplementary file 2 — Additional file 2: Figure S1. Figure displaying the planned stepped-wedge study design. [file 12889_2019_7927_MOESM2_ESM.docx]

Additional file 3: Figure S2. CONSORT diagram


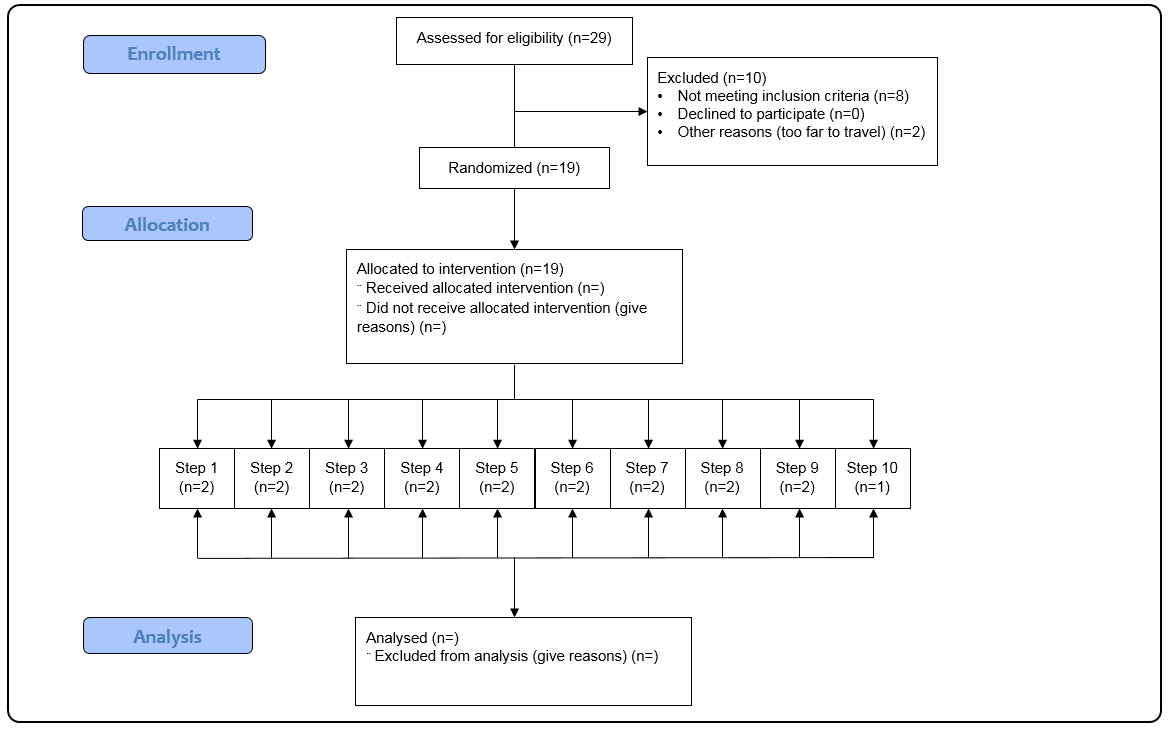

Supplement: Supplementary file 3 — Additional file 3: Figure S2. Figure displaying the enrolment, allocation, and analysis of sites. [file 12889_2019_7927_MOESM3_ESM.docx]
