# Supplementary material for: Increasing the proportion of healthier foods available with and without reducing portion sizes and energy purchased in worksite cafeterias: protocol for a stepped-wedge randomised controlled trial
Source: BMC Public Health. 2019 Dec 2;19:1611. doi: 10.1186/s12889-019-7927-2 (PMC6889705; doi:10.1186/s12889-019-7927-2)
Supplement: Supplementary file 4 — Additional file 4: Figure S3. Figure representing how a range of main meals on a single day may change between baseline and Availability periods. [file 12889_2019_7927_MOESM4_ESM.docx]

Additional file 4: Figure S3. A visual representation of the availability intervention applied to main meals

**Availability**

Below 330kcal

Above 330kcal

**Cheeseburger Minced beef pie**

**Pesto chicken Vegetable chilli**

**Cheeseburger Minced beef pie**

**Breaded pork schnitzel Vegetable chilli**

**Baseline**
