# Supplementary material for: Increasing the proportion of healthier foods available with and without reducing portion sizes and energy purchased in worksite cafeterias: protocol for a stepped-wedge randomised controlled trial
Source: BMC Public Health. 2019 Dec 2;19:1611. doi: 10.1186/s12889-019-7927-2 (PMC6889705; doi:10.1186/s12889-019-7927-2)
Supplement: Supplementary file 5 — Additional file 5. Consent form. [file 12889_2019_7927_MOESM5_ESM.docx]

**Cafeteria study: Consent form**

***Please initial the box next to each statement that you agree to:***

- I confirm that I have read and understand the Information Sheet
- I have had the opportunity to ask questions and had them answered
- I understand that all information regarding worksites will remain confidential and all efforts will be made to ensure they cannot be identified (except as might be required by law)
- I agree that data gathered in this study may be stored anonymously and securely
- I understand that the participation of my worksite is voluntary and that we are free to withdraw at any time without giving a reason

*I agree to take part in this study*

***Signed:****............................................................................................................*

***Date:*** *.................................................................................................................*
